# Visceral adipose tissue and risk of COVID-19 susceptibility, hospitalization, and severity: A Mendelian randomization study

**DOI:** 10.3389/fpubh.2022.1023935

**Published:** 2022-10-21

**Authors:** Lu Chen, Xingang Sun, Deheng Han, Jiawei Zhong, Han Zhang, Liangrong Zheng

**Affiliations:** Department of Cardiology, The First Affiliated Hospital, School of Medicine, Zhejiang University, Hangzhou, China

**Keywords:** COVID-19, visceral adipose tissue, Mendelian randomization, causal associations, risk

## Abstract

**Background:**

Coronavirus Disease 2019 (COVID-19) has rapidly evolved as a global pandemic. Observational studies found that visceral adipose tissue (VAT) increased the likelihood of worse clinical outcomes in COVID-19 patients. Whereas, whether VAT is causally associated with the susceptibility, hospitalization, or severity of COVID-19 remains unconfirmed. We aimed to investigate the causal associations between VAT and susceptibility, hospitalization, and severity of COVID-19.

**Methods:**

We applied a two-sample Mendelian randomization (MR) study to infer causal associations between VAT and COVID-19 outcomes. Single-nucleotide polymorphisms significantly associated with VAT were derived from a large-scale genome-wide association study. The random-effects inverse-variance weighted method was used as the main MR approach, complemented by three other MR methods. Additional sensitivity analyses were also performed.

**Results:**

Genetically predicted higher VAT mass was causally associated with higher risks of COVID-19 susceptibility [odds ratios (ORs) = 1.13; 95% confidence interval (CI), 1.09–1.17; *P* = 4.37 × 10^−12^], hospitalization (OR = 1.51; 95% CI = 1.38–1.65; *P* = 4.14 × 10^−20^), and severity (OR = 1.58; 95% CI = 1.38–1.82; *P* = 7.34 × 10^−11^).

**Conclusion:**

This study provided genetic evidence that higher VAT mass was causally associated with higher risks of susceptibility, hospitalization, and severity of COVID-19. VAT can be a useful tool for risk assessment in the general population and COVID-19 patients, as well as an important prevention target.

## Introduction

Coronavirus Disease 2019 (COVID-19), an infectious disease caused by severe acute respiratory syndrome coronavirus 2 (SARS-CoV-2), has rapidly evolved as a global pandemic (1). By 28, September 2022, a total of 613,410,796 people have been diagnosed with COVID-19 and 6,518,749 people confirmed deaths globally (1). Thus, it is important to recognize risk factors for this disease and determine prevention strategies.

Emerging literature has suggested that obesity is correlated with the risk, severity, and prognosis of COVID-19 (2–5). In these studies, obesity was commonly assessed by body mass index (BMI), estimated by the ratio of weight over height. However, since obesity is a heterogeneous disorder, there are considerable individual variations in the body fat distribution and metabolic profile even with the same BMI (6). Visceral adipose tissue (VAT) is the adiposity accumulated around abdominal viscera, which can be measured by several imaging technologies, including magnetic resonance imaging, computed tomography (CT), and dual-energy X-ray absorptiometry (DXA) (7). VAT is considered as a marker of ectopic fat deposition and disturbed hormonal milieu, and is metabolically more active compared with subcutaneous adipose tissue (8). Recently, several observational studies found that VAT increased the likelihood of worse clinical outcomes in COVID-19 patients (9–11). Whereas, the current data seldom investigated the relationship between VAT and susceptibility and hospitalization of COVID-19. In addition, the causal associations between VAT and COVID-19 outcomes remain unconfirmed.

Mendelian randomization (MR) is a widely used epidemiology method with genetic variants utilized as instrumental variables (IVs) to perform causal inferences between exposures (risk factors) and outcomes (diseases) (12). Since observational studies are prone to the possibility of unmeasured confounders and reverse causation, the observed exposure-outcome association may not reflect an accurate causal relationship (13). Besides, although randomized controlled trials (RCTs) can demonstrate causality with high evidence level, well-designed RCTs are often expensive and time-consuming, and not always ethical (14). As genetic variants are randomly and independently inherited at conception and prior to the development of diseases, MR study provides a natural experiment simulating an RCT but avoids confounding from environmental effects and reverse causality. In this study, we aimed to apply two-sample MR analyses to infer the causal effects of VAT on susceptibility, hospitalization, and severity of COVID-19.

## Methods

### Study design

**Figure 1:**
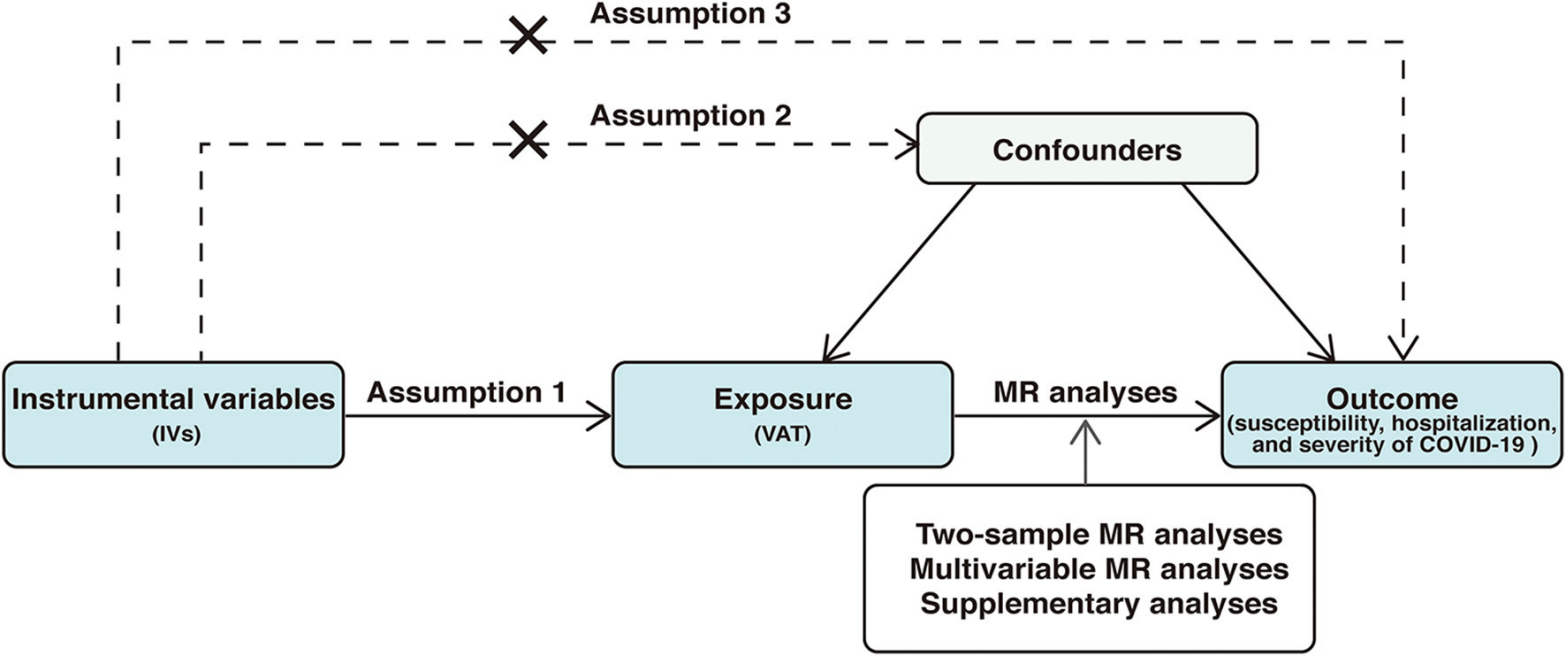
Overview of the study design. The study was based on 3 important assumptions: 1, genetic variants selected as instrumental variables (IVs) should be significantly associated with the exposure (VAT), 2, IVs should not be associated with any confounders, and 3, IVs should not affect the outcome (COVID-19 outcomes) through any other pathways except the exposure (VAT). VAT, visceral adipose tissue; MR, Mendelian randomization; COVID-19, Coronavirus Disease 2019.

The present study used summary data from a large-scale genome-wide association study (GWAS) for VAT and the COVID19 Host Genetics Initiative for COVID-19 outcomes. The study was based on 3 important assumptions: (1) genetic variants selected as IVs should be significantly associated with the exposure (VAT), (2) IVs should not be associated with any confounders, and (3) IVs should not affect the outcome (COVID-19 outcomes) through any other pathways except the exposure (VAT). We first applied a two-sample MR study to infer causal associations between VAT and COVID-19 outcomes. Then, we conducted multivariable MR (MVMR) analyses to investigate the direct effect of VAT on COVID-19 outcomes. Supplementary analyses were also performed to minimize the bias caused by population stratification and sample overlap. The overview of the study design was displayed in Figure 1.

### VAT data sources

Summary data for VAT were derived from a recent large-scale GWAS performed by Karlsson et al. (15). This study used two sub-cohorts to predict VAT in UK Biobank, with VAT-training dataset measured by DXA used to create prediction models and VAT-application dataset used to calculate VAT according to the prediction models. Overall, the training dataset involved 4,198 participants and the application dataset involved 325,153 participants. We selected single-nucleotide polymorphisms (SNPs) significantly associated with VAT mass at the genome-wide significance level (*P* < 5 × 10^−8^) and further pruned those SNPs for independence (*r*^2^ = 0.001; >10,000 kb) by “clump_data” function in “TwosampleMR” package. Finally, we obtained 221 VAT-associated SNPs which were used as IVs in further analyses (Supplementary Table 1).

### Outcome data sources

As shown in Table 1, the summary-level data for COVID-19 outcomes (including susceptibility, hospitalization, and severity of COVID-19) were obtained from the COVID19 Host Genetics Initiative (round 6, released on June 15, 2021, https://www.covid19hg.org/results/r6/), an ongoing initiative which aims to facilitate COVID-19 genetic host research through bringing together the human genetics data. To study the risk of COVID-19 susceptibility, we downloaded the GWAS meta-analysis of “Covid vs. population” (C2_ALL_leave_23andme), in which cases were defined as individuals who reported SARS-CoV-2 infection regardless of symptoms and controls were defined as non-cases (16). This GWAS meta-analysis involved 112,612 cases and 2,474,079 controls. For COVID-19 hospitalization, we downloaded the GWAS meta-analysis of “Hospitalized covid vs. population” (B2_ALL_leave_23andme), with cases defined as SARS-CoV-2 infected individuals who were hospitalized due to COVID-19-related symptoms and controls defined as non-cases (16). There were 24,274 cases and 2,061,529 controls in this GWAS. Additionally, we downloaded the GWAS meta-analysis of “Very severe respiratory confirmed covid vs. population” (A2_ALL_leave_23andme) for COVID-19 severity. This GWAS meta-analysis consisted of 8,779 cases and 1,001,875 controls, with cases defined as SARS-CoV-2 infected individuals who required respiratory support or had died and controls defined as non-cases (16). If the SNPs were unavailable in the outcome datasets, we searched for proxy SNPs in high linkage disequilibrium (*r*^2^ > 0.8) by using an online website (http://snipa.helmholtz-muenchen.de/snipa3/) (Supplementary Table 2).

**Table 1:** Characteristics of the data sources for COVID-19 outcomes.

**Phenotype**	**Traits**	**Ancestry**	**Cases**	**Controls**	**UKB**	**Sample** overlap^*^	**Use**	**Release (URL)**
C2: Reported SARS-CoV-2 infection vs. population controls	COVID-19 susceptibility	Mixed	112,612	2,474,079	441,331	≤ 12.6%	Primary analyses	r6 (https://www.covid19hg.org/results/r6/)
B2: Hospitalized COVID19+ vs. population controls	COVID-19 hospitalization	Mixed	24,274	2,061,529	436,043	≤ 15.6%	Primary analyses	r6 (https://www.covid19hg.org/results/r6/)
A2: Critically ill COVID19+ vs. population controls	COVID-19 severity	Mixed	8,779	1,001,875	420,531	≤ 32.2%	Primary analyses	r6 (https://www.covid19hg.org/results/r6/)
C2: Reported SARS-CoV-2 infection vs. population controls	COVID-19 susceptibility (European, leave UKB)	European	74,614	1,803,529	0	0	Supplementary analyses	r6 (https://www.covid19hg.org/results/r6/)
B2: Hospitalized COVID19+ vs. population controls	COVID-19 hospitalization (European, leave UKB)	European	14,925	1,393,029	0	0	Supplementary analyses	r6 (https://www.covid19hg.org/results/r6/)
A2: Critically ill COVID19+ vs. population controls	COVID-19 severity (European, leave UKB)	European	4,297	374,224	0	0	Supplementary analyses	r5 (https://www.covid19hg.org/results/r5/)

COVID-19, Coronavirus Disease 2019; UKB, UK Biobank.

* The overlapping sample size (assuming that all of the overlapping samples appeared in the cohorts of exposure and outcome) was divided by the larger sample size of the corresponding outcome trait and exposure.

### Statistical analysis

In the primary MR analyses, we applied the random-effects inverse-variance weighted (IVW) method as the main MR approach to obtain causal estimates between VAT and COVID-19 outcomes. We also performed other three MR methods to test the stability of the IVW results, including weighted median, MR-Egger, and MR-pleiotropy residual sum and outlier (MR-PRESSO). The weighted median method generates robust and consistent causal estimates, assuming that more than 50% of the weights are derived from valid SNPs. The MR-Egger was performed to evaluate horizontal pleiotropy with *P*-value for its intercept and provide pleiotropy-corrected estimates if significant horizontal pleiotropy was detected by the intercept test; however, this model usually consumes statistical power. The MR-PRESSO method can identify outliers and generate causal estimates after removing the outliers. For sensitivity analyses, we used the Cochran's *Q*-statistic and *I*^2^ and *P*-value for the intercept in MR-Egger to detect heterogeneity (*P*__*Q*_−statistics_ < 0.05 or *I*^2^ > 25%) and horizontal pleiotropy (*P*_intercept_ < 0.05). We also performed the leave-one-out analysis to investigate whether the association was driven by any single SNP. Regression-based MVMR analyses were further conducted to investigate the direct effect of VAT on COVID-19 outcomes, independent from the effect of BMI which was highly correlated with VAT. Genetic associations of instruments with BMI were obtained from publicly available GWAS (17). We used the mRnd (http://cnsgenomics.com/shiny/mRnd/), a web application, to estimate the required OR of the exposure on the outcome to achieve 80% statistical power.

As there was some sample overlap (UK Biobank) between the GWAS of VAT and COVID-19 outcomes and the ancestries of participants in COVID-19 outcomes were mixed (Table 1), we further performed supplementary analyses by utilizing European-ancestry specific summary statistics which also leaves UK Biobank cohort out to minimize the bias caused by population stratification and sample overlap. Detailed information was presented in Table 1.

Results were reported as odds ratios (ORs) and corresponding 95% confidence intervals (CIs) of COVID-19 outcomes which were scaled to one standard deviation (SD) increase in genetically predicted VAT mass. Accounting for three outcomes included in the study, we set *P*-values < 0.017 (0.05/3) as statistical significance. Associations with *P*-values between 0.05 and 0. 017 were regarded as suggestive associations. No ethical approval was required as this study used summary-level data from the published studies and publicly available GWASs. We performed MR analyses in R version 4.0.2 using the R packages TwosampleMR (18), MR-PRESSO (19), and MendelianRandomization (20).

## Results

A total of 221 SNPs were identified as IVs for predicted VAT mass. These SNPs explained ~3.70% variance in VAT mass. The F-statistics for each SNP ranged from 29.72 to 658.12, reflecting a low possibility of weak instruments. There was >80% statistical power to detect significant differences at an OR of 1.05 or higher for COVID-19 susceptibility, 1.10 or higher for COVID-19 hospitalization, and 1.16 or higher for COVID-19 severity (Supplementary Table 3).

**Figure 2:**
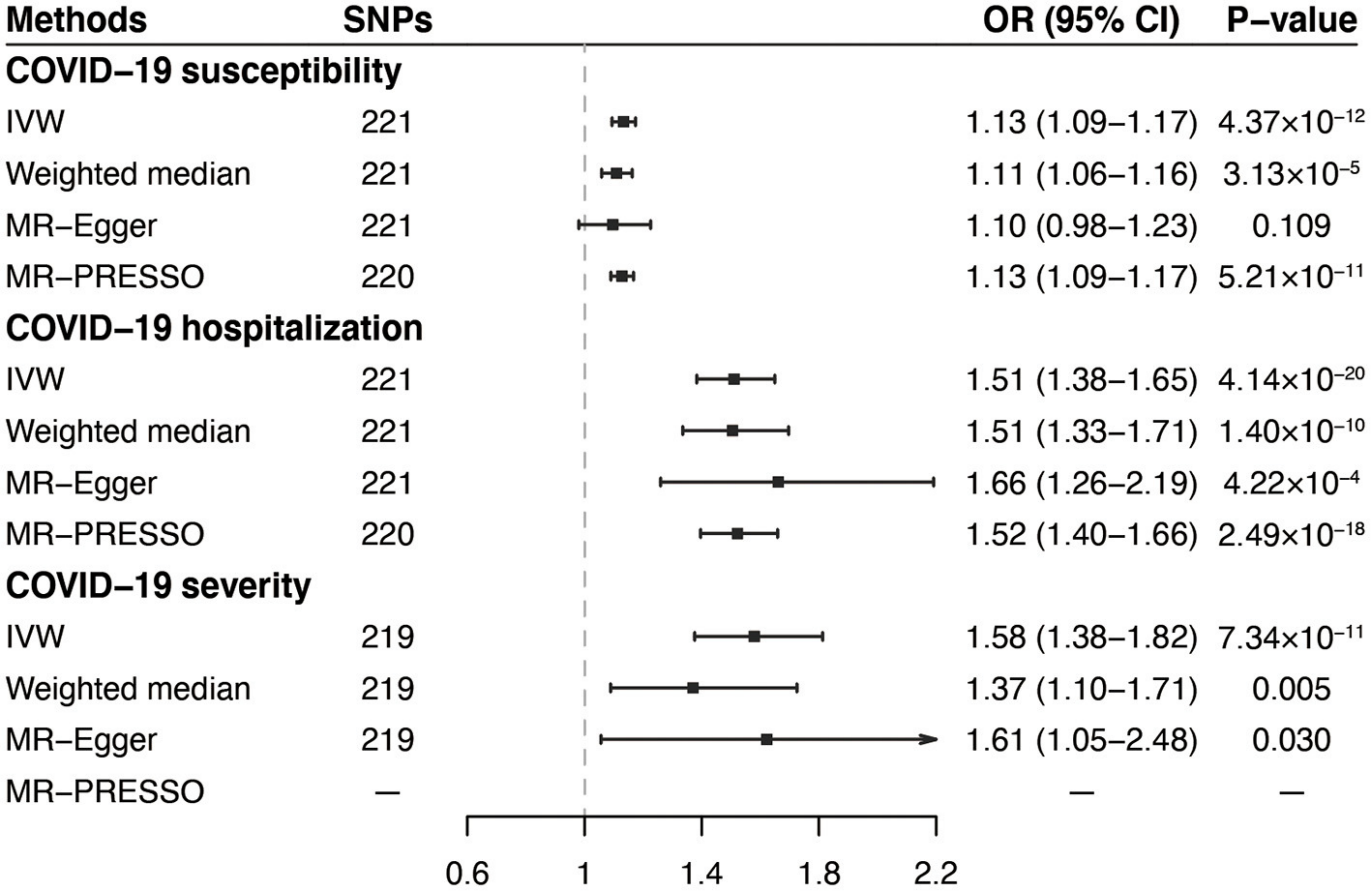
Associations of genetically predicted VAT mass with COVID-19 outcomes in primary analyses. VAT, visceral adipose tissue; COVID-19, Coronavirus Disease 2019; SNPs, single nucleotide polymorphisms; OR, odds ratio; CI, confidence interval; IVW, inverse-variance weighted; MR-PRESSO, MR-pleiotropy residual sum and outlier.

In the standard IVW analyses, as shown in Figure 2, we observed strong evidence of causal associations of the increased VAT mass with higher risks of COVID-19 susceptibility (OR = 1.13; 95% CI, 1.09–1.17; *P* = 4.37 × 10^−12^), COVID-19 hospitalization (OR = 1.51; 95% CI = 1.38–1.65; *P* = 4.14 × 10^−20^), and COVID-19 severity (OR = 1.58; 95% CI = 1.38–1.82; *P* = 7.34 × 10^−11^). Results from the weighted median and MR-Egger methods were robust and consistent, except for COVID-19 susceptibility and severity in MR-Egger, which showed a similar pattern of effect but with broader CIs (Figure 2). The intercept term in MR-Egger regression suggested no significant overall horizontal pleiotropy (all *P*_intercept_ > 0.05; Table 2). However, there was some evidence of heterogeneity for COVID-19 susceptibility and hospitalization measured by Cochran *Q* and *I*^2^ (*P*__*Q*_−statistics_ = 1.61 × 10^−4^, *I*^2^ = 27.5% for COVID-19 susceptibility, and *P*__*Q*_−statistics_ = 0.006, *I*^2^ = 20.4% for COVID-19 hospitalization, respectively; Table 2). MR-PRESSO outlier test detected one outlier (rs13135092) in the analyses for COVID-19 susceptibility and one outlier (rs1446585) in the analyses for COVID-19 hospitalization. Whereas, after excluding these outliers, the results still indicated that genetically predicted increased VAT was associated with higher risks of COVID-19 susceptibility and hospitalization. The leave-one-out analysis further illustrated that the MR estimates were not driven by any individual SNP.

**Table 2:** Heterogeneity and pleiotropy assessment in primary analyses.

**Outcomes**	**SNPs**	***P*__*Q*_−statistics_**	***I*** **^2^ (%)**	***P*** **_intercept_**	***P*** **_MR − PRESSO global test_**	**Outlier**
COVID-19 susceptibility	221	1.61 × 10^−4^	27.5	0.535	<0.001	rs13135092
COVID-19 hospitalization	221	0.006	20.5	0.488	0.008	rs1446585
COVID-19 severity	219	0.428	1.4	0.923	0.431	–

SNPs, single nucleotide polymorphisms; MR-PRESSO, Mendelian randomization pleiotropy residual sum and outlier; COVID-19, Coronavirus Disease 2019.

**Figure 3:**
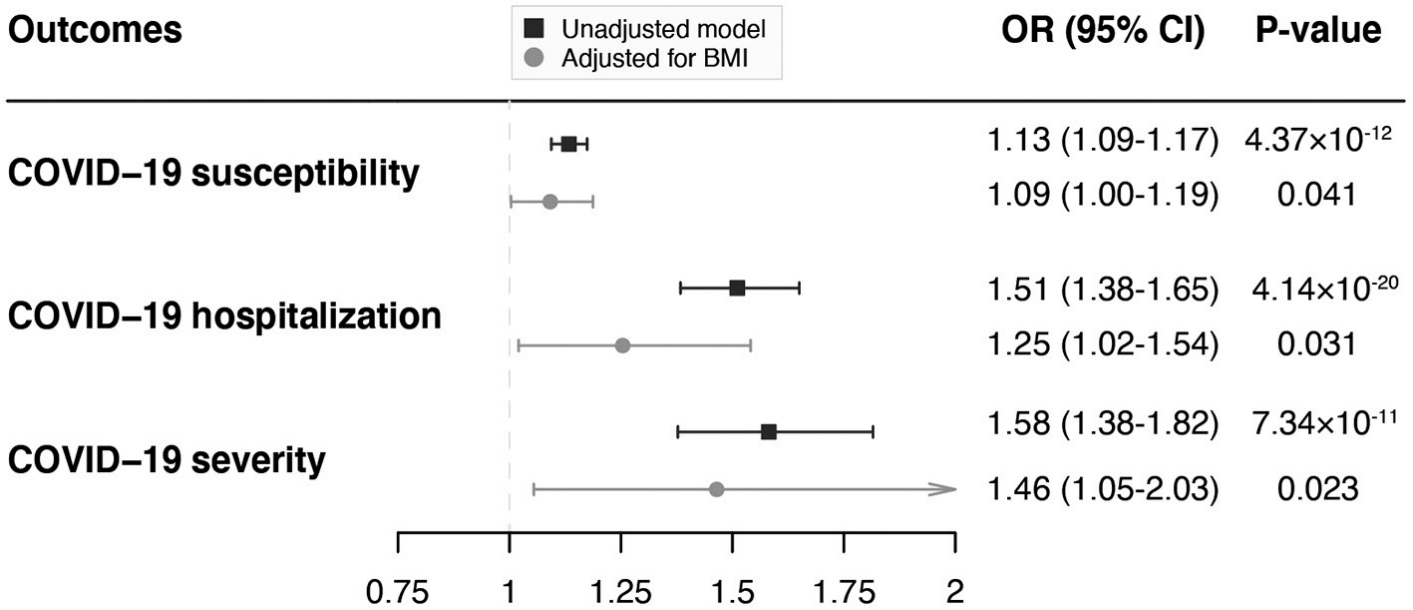
MVMR analyses of the associations of genetically predicted VAT mass with COVID-19 outcomes adjusting for BMI. MVMR, multivariable Mendelian randomization; VAT, visceral adipose tissue; COVID-19, Coronavirus Disease 2019; BMI, body mass index; OR, odds ratio; CI, confidence interval.

According to the MVMR analyses adjusting for BMI, although the MR estimates were attenuated, there was still suggestive evidence of associations of VAT with COVID-19 susceptibility (OR = 1.09; 95% CI, 1.00–1.19; *P* = 0.041), hospitalization (OR = 1.25; 95% CI, 1.02–1.54; *P* = 0.031), and severity (OR = 1.46; 95% CI, 1.05–2.03; *P* = 0.023; Figure 3), indicating that the effect of VAT on COVID-19 outcomes were independent of the effect from BMI.

When performing the supplementary analyses by utilizing COVID-19 GWAS summary statistics constrained to European ancestry and excluding the UK Biobank cohort, the results were generally consistent and stable across all MR methods, although weakened associations were observed for COVID-19 severity in the weighted median method and for both COVID-19 hospitalization and severity in MR-Egger method (Supplementary Figure 1). We found no evidence of horizontal pleiotropy indicated by intercepts and modest heterogeneity in the analyses for COVID-19 susceptibility and hospitalization (Supplementary Table 4). The leave-one-out analysis confirmed that the results were stable when eliminating the SNPs one by one (Supplementary Table 5).

## Discussion

In this two-sample MR study, we found that genetically predicted higher VAT was associated with increased risks of COVID-19 susceptibility, hospitalization, and severity. The associations remained in the MVMR analyses adjusting for BMI and supplementary analyses.

Recent studies have suggested that VAT was correlated with adverse outcomes in COVID-19 patients (9–11, 21, 22). The relationship between VAT and COVID-19 was firstly investigated by Petersen et al. (10) in a cross-sectional pilot study involving 30 patients with COVID-19. They found that VAT specifically increased the likelihood of severe courses of COVID-19. This finding was confirmed by later studies. Yang et al. (23) conducted a retrospective study of 143 COVID-19 patients to assess the association between abdominal adipose tissue distribution and the severity of COVID-19, which suggested that patients with higher VAT were more likely to develop critical illness. In another retrospective study with 51 COVID-19 infected patients undergoing abdominopelvic CT, Chandarana et al. (9) observed higher VAT in COVID-19 patients that required hospitalization compared with those outpatients. Furthermore, the addition of VAT to the clinical model significantly improved its performance for identifying patients requiring hospitalization (9). Similarly, Favre et al. (11) identified high visceral fat amount as a stronger predictor of COVID-19 severity in European adults compared with BMI.

However, most previous observational studies were retrospective and conducted in single-center, with relatively small sample sizes. The possibility of selection bias, unmeasured confounding, and reverse causation should also be considered. Thus, large-scale studies are warranted to confirm these results. Utilizing summary-level data from large-scale GWASs, this MR study confirmed that genetically predicted higher VAT was associated with elevated risks of susceptibility, hospitalization, and severity of COVID-19, largely consistent with the results of previous studies.

Several VAT-related biological mechanisms may explain the observed causal associations. It was reported that individuals with greater levels of VAT had higher levels of adiposity-related inflammation (24). The infiltration of macrophages among hypertrophied adipocytes leads to elevated secretion of inflammatory cytokines, including tumor necrosis factor *α* and interleukin 6, and decreased production of adiponectin, a protective adipokine (25). Among COVID-19^+^ VAT, Colleluori et al. (26) detected a higher prevalence of CD68^+^ macrophages, which supported the presence of higher local VAT inflammation. In addition, angiotensin-converting enzyme 2 receptor was found to be highly expressed in VAT and may play a role in increasing the viral entry of SARS-CoV-2 (27, 28). Furthermore, VAT was correlated with many health and metabolic abnormalities, such as brain health, type 2 diabetes, cardiovascular and respiratory diseases, and cancers (29), and previous studies have demonstrated associative evidence between many of these conditions and COVID-19 (30–32).

The current results have implications for clinical practice. Since genetically predicted higher VAT was correlated with increased risks of COVID-19 susceptibility, hospitalization, and severity, VAT might be a useful and important tool for risk assessment in the general population and COVID-19 patients. As many hospitalized COVID-19 patients undergo chest or abdominal CT scans for the purpose of diagnosis or clinical care, these CTs can be used to quantify VAT but with no additional costs or radiation exposure. Besides, the reduction of VAT through aerobic exercise and caloric restriction may play a role in reducing the risk of getting infected and developing severe courses of COVID-19 (33, 34), which warrants further investigation. Moreover, a recent prospective study found that a higher baseline BMI was correlated with a reduced adaptive response to the COVID-19 mRNA vaccine; however, weight loss seemed effective at reversing this negative effect in subjects with obesity (35). Further research is also needed to evaluate the impact of VAT reduction on COVID-19 susceptibility by affecting the immune response to the COVID-19 vaccine.

The major strength of this study is that we applied the MR method to infer the causal associations between VAT and the risks of COVID-19 susceptibility, hospitalization, and severity, utilizing summary-level data derived from large-scale GWASs, which avoids residual confounding and reverse causality. Besides, in order to minimize the bias caused by population stratification and sample overlap, we conducted supplementary analyses utilizing European-ancestry-specific summary statistics and excluding the UK Biobank cohort. However, our study still has several limitations. First, we did not perform age or sex-stratified analyses due to a lack of available summary-level data for COVID-19. Second, pleiotropy is a major issue for any MR study. Nonetheless, we detected no significant overall horizontal pleiotropy from the intercept term in MR-Egger regression. In addition, the causal associations remained consistent after excluding the outliers and adjusting for BMI in the MVMR analyses. Third, since MR studies are vulnerable to selection bias due to inevitably recruiting participants surviving the genetic instruments and competing for risk of COVID-19 (36), further investigations are needed to confirm the results of this study.

## Conclusion

In conclusion, this MR study provided genetic evidence that higher VAT was associated with increased risks of COVID-19 susceptibility, hospitalization, and severity, highlighting that VAT can be a useful and important tool for risk assessment in the general population and COVID-19 patients and VAT management may play a pivotal role in reducing the risk of getting infected and developing severe courses of COVID-19.

## Data availability statement

The original contributions presented in the study are included in the article/Supplementary material, further inquiries can be directed to the corresponding author.

## Author contributions

LC and LZ designed the study. LC, XS, DH, JZ, and HZ performed the data analysis and interpreted the data. All authors were involved in writing the paper and had final approval of the submitted and published versions.

## Funding

This study was supported by the National Natural Science Foundation of China (No. 81873484).

## Conflict of interest

The authors declare that the research was conducted in the absence of any commercial or financial relationships that could be construed as a potential conflict of interest.

## Publisher's note

All claims expressed in this article are solely those of the authors and do not necessarily represent those of their affiliated organizations, or those of the publisher, the editors and the reviewers. Any product that may be evaluated in this article, or claim that may be made by its manufacturer, is not guaranteed or endorsed by the publisher.
