# Supplementary material for: Visceral adipose tissue and risk of COVID-19 susceptibility, hospitalization, and severity: A Mendelian randomization study
Source: Front Public Health. 2022 Oct 21;10:1023935. doi: 10.3389/fpubh.2022.1023935 (PMC9634527; doi:10.3389/fpubh.2022.1023935)
Supplement: Supplementary file 1 [file Data_Sheet_1.DOCX]

**Figure S1.** Associations of genetically predicted VAT mass with COVID-19 outcomes in supplementary analyses.

**
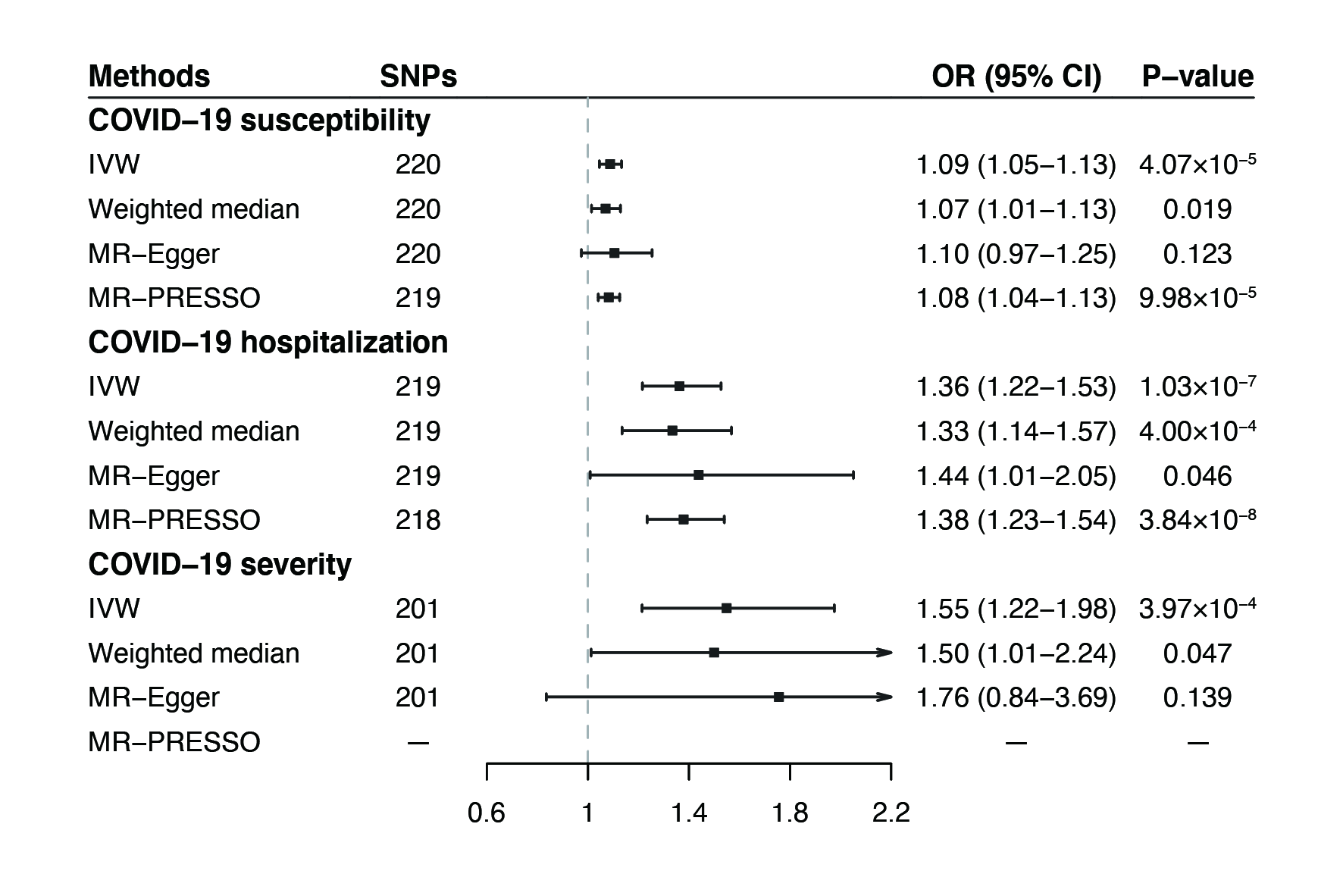

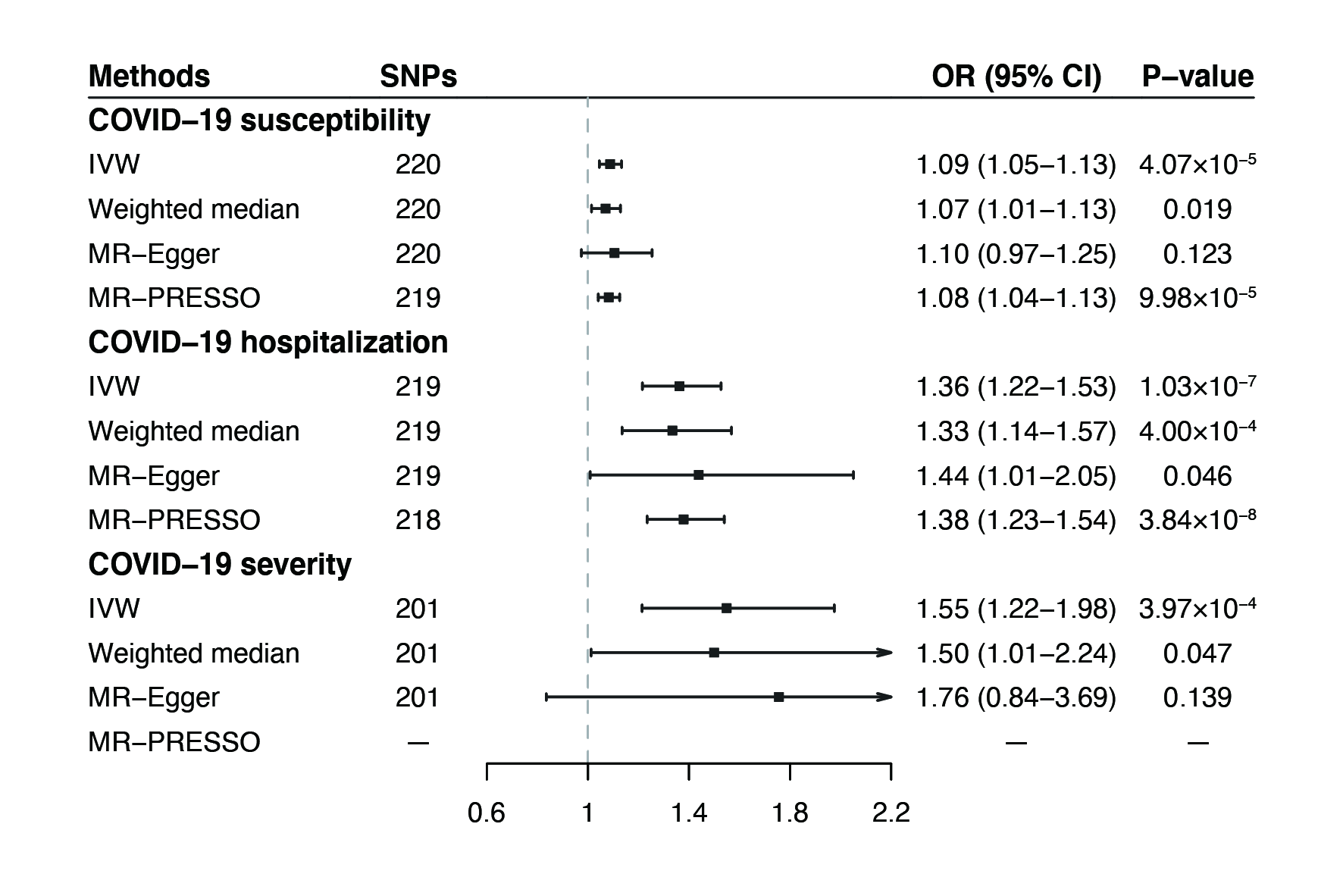
**

Abbreviations: VAT, visceral adipose tissue; COVID-19, Coronavirus Disease 2019; SNPs, single nucleotide polymorphisms; OR, odds ratio; CI, confidence interval; IVW, inverse-variance weighted; MR-PRESSO, MR-pleiotropy residual sum and outlier.

**Table S1.** Information on instrumental variables utilized in this Mendelian randomization study.

| **SNP** | **EA** | **NEA** | **EAF** | **R^2^** | **F** | **Beta** | **SE** | **P-value** |
| --- | --- | --- | --- | --- | --- | --- | --- | --- |
| rs56094641 | G | A | 0.771 | 2.02E-03 | 658.12 | 0.065 | 0.003 | 3.82E-145 |
| rs538656 | T | G | 0.724 | 7.42E-04 | 241.56 | 0.045 | 0.003 | 1.79E-54 |
| rs13393304 | A | G | 0.877 | 5.66E-04 | 184.11 | -0.044 | 0.003 | 6.12E-42 |
| rs539515 | C | A | 0.805 | 4.76E-04 | 154.95 | 0.038 | 0.003 | 1.43E-35 |
| rs62262093 | T | C | 0.365 | 4.49E-04 | 145.93 | -0.030 | 0.002 | 1.34E-33 |
| rs11030112 | A | G | 0.756 | 4.30E-04 | 140.00 | 0.031 | 0.003 | 2.65E-32 |
| rs76111507 | T | C | 0.989 | 4.05E-04 | 131.72 | -0.075 | 0.007 | 1.72E-30 |
| rs10938398 | A | G | 0.675 | 3.94E-04 | 128.22 | 0.028 | 0.003 | 1.00E-29 |
| rs72892910 | T | G | 0.820 | 3.68E-04 | 119.73 | 0.036 | 0.003 | 7.24E-28 |
| rs7498665 | G | A | 0.739 | 3.48E-04 | 113.32 | 0.027 | 0.003 | 1.83E-26 |
| rs4808762 | C | T | 0.791 | 3.44E-04 | 112.01 | 0.029 | 0.003 | 3.55E-26 |
| rs10182458 | G | A | 0.441 | 3.41E-04 | 111.00 | 0.026 | 0.002 | 5.90E-26 |
| rs4402589 | T | G | 0.454 | 3.41E-04 | 110.92 | -0.026 | 0.002 | 6.17E-26 |
| rs10423928 | A | T | 0.828 | 3.38E-04 | 109.93 | -0.033 | 0.003 | 1.01E-25 |
| rs2307111 | C | T | 0.384 | 3.24E-04 | 105.25 | -0.026 | 0.003 | 1.08E-24 |
| rs7132908 | A | G | 0.748 | 3.09E-04 | 100.49 | 0.026 | 0.003 | 1.19E-23 |
| rs9471333 | C | T | 0.548 | 2.96E-04 | 96.16 | 0.024 | 0.002 | 1.06E-22 |
| rs113211479 | A | G | 0.664 | 2.88E-04 | 93.69 | 0.024 | 0.003 | 3.70E-22 |
| rs3784692 | C | T | 0.463 | 2.85E-04 | 92.83 | -0.024 | 0.003 | 5.70E-22 |
| rs669696 | A | C | 0.714 | 2.85E-04 | 92.68 | -0.024 | 0.003 | 6.15E-22 |
| rs17770336 | T | C | 0.790 | 2.69E-04 | 87.64 | 0.025 | 0.003 | 7.85E-21 |
| rs10740991 | G | C | 0.854 | 2.66E-04 | 86.60 | 0.026 | 0.003 | 1.33E-20 |
| rs1454687 | C | G | 0.453 | 2.66E-04 | 86.38 | 0.023 | 0.002 | 1.49E-20 |
| rs71658797 | A | T | 0.971 | 2.58E-04 | 84.06 | 0.035 | 0.004 | 4.79E-20 |
| rs9320823 | T | C | 0.689 | 2.58E-04 | 84.01 | -0.023 | 0.003 | 4.92E-20 |
| rs7156625 | A | G | 0.805 | 2.50E-04 | 81.25 | 0.027 | 0.003 | 1.99E-19 |
| rs6096886 | G | A | 0.827 | 2.49E-04 | 80.92 | -0.028 | 0.003 | 2.35E-19 |
| rs6739755 | A | G | 0.708 | 2.46E-04 | 79.89 | 0.023 | 0.003 | 3.95E-19 |
| rs429358 | C | T | 0.849 | 2.45E-04 | 79.61 | -0.030 | 0.003 | 4.57E-19 |
| rs2678204 | G | T | 0.745 | 2.39E-04 | 77.76 | 0.023 | 0.003 | 1.16E-18 |
| rs9358912 | T | G | 0.634 | 2.39E-04 | 77.67 | -0.025 | 0.003 | 1.22E-18 |
| rs2304608 | A | C | 0.701 | 2.36E-04 | 76.78 | 0.030 | 0.003 | 1.91E-18 |
| rs35060985 | A | G | 0.701 | 2.34E-04 | 76.23 | 0.023 | 0.003 | 2.52E-18 |
| rs1591726 | T | C | 0.589 | 2.18E-04 | 70.75 | 0.022 | 0.003 | 4.06E-17 |
| rs1652376 | T | G | 0.390 | 2.17E-04 | 70.70 | -0.021 | 0.002 | 4.17E-17 |
| rs1225060 | A | G | 0.835 | 2.14E-04 | 69.44 | 0.023 | 0.003 | 7.88E-17 |
| rs62190394 | T | C | 0.724 | 2.08E-04 | 67.59 | 0.022 | 0.003 | 2.02E-16 |
| rs76040172 | A | G | 0.935 | 2.06E-04 | 67.08 | -0.045 | 0.006 | 2.60E-16 |
| rs2253310 | C | G | 0.470 | 2.05E-04 | 66.70 | -0.021 | 0.003 | 3.16E-16 |
| rs62084234 | G | A | 0.703 | 2.04E-04 | 66.40 | 0.025 | 0.003 | 3.68E-16 |
| rs117151227 | C | T | 0.977 | 2.02E-04 | 65.82 | -0.062 | 0.008 | 4.95E-16 |
| rs62261725 | G | A | 0.711 | 2.02E-04 | 65.60 | -0.021 | 0.003 | 5.52E-16 |
| rs12477088 | C | T | 0.533 | 2.01E-04 | 65.38 | -0.020 | 0.003 | 6.17E-16 |
| rs2285640 | G | A | 0.592 | 2.00E-04 | 64.97 | 0.020 | 0.002 | 7.58E-16 |
| rs4239060 | A | G | 0.865 | 1.99E-04 | 64.64 | -0.026 | 0.003 | 8.98E-16 |
| rs879620 | C | T | 0.344 | 1.99E-04 | 64.63 | -0.021 | 0.003 | 9.02E-16 |
| rs2744973 | T | C | 0.591 | 1.94E-04 | 63.05 | 0.021 | 0.003 | 2.01E-15 |
| rs10756714 | G | A | 0.622 | 1.91E-04 | 62.05 | -0.020 | 0.002 | 3.35E-15 |
| rs7550711 | T | C | 0.988 | 1.88E-04 | 61.18 | 0.062 | 0.008 | 5.20E-15 |
| rs9989141 | C | T | 0.506 | 1.87E-04 | 60.66 | -0.020 | 0.003 | 6.77E-15 |
| rs62477685 | T | A | 0.554 | 1.86E-04 | 60.39 | -0.019 | 0.003 | 7.78E-15 |
| rs13017207 | A | G | 0.716 | 1.85E-04 | 60.07 | -0.020 | 0.003 | 9.17E-15 |
| rs13062093 | G | T | 0.632 | 1.82E-04 | 59.09 | 0.020 | 0.003 | 1.51E-14 |
| rs4482463 | C | A | 0.778 | 1.81E-04 | 58.88 | 0.036 | 0.005 | 1.68E-14 |
| rs8015400 | C | A | 0.553 | 1.74E-04 | 56.65 | -0.020 | 0.003 | 5.22E-14 |
| rs11880870 | G | A | 0.321 | 1.72E-04 | 55.78 | -0.018 | 0.002 | 8.09E-14 |
| rs55726687 | A | G | 0.848 | 1.72E-04 | 55.78 | 0.023 | 0.003 | 8.12E-14 |
| rs1928496 | C | T | 0.746 | 1.71E-04 | 55.51 | -0.021 | 0.003 | 9.30E-14 |
| rs10896012 | C | T | 0.863 | 1.71E-04 | 55.48 | 0.022 | 0.003 | 9.46E-14 |
| rs9522285 | A | G | 0.716 | 1.70E-04 | 55.44 | 0.019 | 0.003 | 9.61E-14 |
| rs113866544 | C | T | 0.926 | 1.69E-04 | 55.01 | 0.037 | 0.005 | 1.20E-13 |
| rs66679256 | T | C | 0.556 | 1.69E-04 | 54.87 | 0.018 | 0.002 | 1.29E-13 |
| rs40067 | A | G | 0.738 | 1.67E-04 | 54.46 | -0.024 | 0.003 | 1.59E-13 |
| rs3843540 | C | T | 0.598 | 1.66E-04 | 53.85 | -0.026 | 0.003 | 2.17E-13 |
| rs11150745 | G | A | 0.790 | 1.63E-04 | 53.14 | -0.019 | 0.003 | 3.11E-13 |
| rs7845090 | G | A | 0.641 | 1.63E-04 | 53.09 | 0.020 | 0.003 | 3.19E-13 |
| rs653958 | G | A | 0.684 | 1.63E-04 | 53.03 | 0.019 | 0.003 | 3.28E-13 |
| rs62104473 | T | C | 0.832 | 1.63E-04 | 52.90 | 0.019 | 0.003 | 3.50E-13 |
| rs55742087 | T | C | 0.847 | 1.62E-04 | 52.84 | -0.023 | 0.003 | 3.61E-13 |
| rs13135092 | G | A | 0.975 | 1.58E-04 | 51.46 | 0.033 | 0.005 | 7.31E-13 |
| rs2481665 | C | T | 0.813 | 1.58E-04 | 51.33 | -0.018 | 0.002 | 7.82E-13 |
| rs9277979 | T | C | 0.882 | 1.57E-04 | 51.10 | 0.023 | 0.003 | 8.77E-13 |
| rs12459368 | G | A | 0.694 | 1.56E-04 | 50.58 | -0.020 | 0.003 | 1.15E-12 |
| rs9641499 | A | C | 0.603 | 1.55E-04 | 50.49 | -0.018 | 0.003 | 1.20E-12 |
| rs577525 | T | C | 0.403 | 1.54E-04 | 50.02 | -0.018 | 0.002 | 1.52E-12 |
| rs72663503 | T | C | 0.882 | 1.53E-04 | 49.86 | 0.021 | 0.003 | 1.65E-12 |
| rs1834144 | A | C | 0.555 | 1.53E-04 | 49.78 | -0.018 | 0.003 | 1.72E-12 |
| rs13337177 | T | G | 0.791 | 1.52E-04 | 49.47 | -0.023 | 0.003 | 2.02E-12 |
| rs245775 | A | G | 0.759 | 1.52E-04 | 49.33 | -0.020 | 0.003 | 2.16E-12 |
| rs76327888 | T | G | 0.710 | 1.50E-04 | 48.75 | 0.023 | 0.003 | 2.90E-12 |
| rs4929923 | T | C | 0.533 | 1.48E-04 | 48.24 | -0.018 | 0.003 | 3.78E-12 |
| rs2926614 | T | C | 0.764 | 1.48E-04 | 48.05 | -0.022 | 0.003 | 4.16E-12 |
| rs56356382 | C | T | 0.795 | 1.45E-04 | 47.17 | -0.022 | 0.003 | 6.51E-12 |
| rs61910767 | T | C | 0.946 | 1.45E-04 | 47.04 | -0.023 | 0.003 | 6.95E-12 |
| rs719802 | T | C | 0.493 | 1.44E-04 | 46.81 | 0.017 | 0.003 | 7.84E-12 |
| rs7893571 | G | T | 0.796 | 1.42E-04 | 46.03 | -0.018 | 0.003 | 1.17E-11 |
| rs4073582 | A | G | 0.834 | 1.41E-04 | 45.97 | -0.018 | 0.003 | 1.20E-11 |
| rs3787075 | G | C | 0.695 | 1.41E-04 | 45.87 | 0.018 | 0.003 | 1.26E-11 |
| rs35697587 | G | A | 0.407 | 1.40E-04 | 45.39 | 0.017 | 0.002 | 1.62E-11 |
| rs7982447 | C | T | 0.702 | 1.40E-04 | 45.38 | 0.021 | 0.003 | 1.62E-11 |
| rs7649970 | T | C | 0.880 | 1.39E-04 | 45.20 | 0.025 | 0.004 | 1.78E-11 |
| rs2172131 | T | C | 0.706 | 1.39E-04 | 45.12 | 0.017 | 0.003 | 1.85E-11 |
| rs145350287 | A | T | 0.990 | 1.39E-04 | 45.06 | -0.042 | 0.006 | 1.92E-11 |
| rs61813293 | T | G | 0.953 | 1.39E-04 | 45.04 | 0.024 | 0.004 | 1.93E-11 |
| rs3943933 | A | T | 0.427 | 1.38E-04 | 44.80 | 0.017 | 0.002 | 2.18E-11 |
| rs4558773 | A | G | 0.716 | 1.38E-04 | 44.75 | 0.017 | 0.003 | 2.24E-11 |
| rs2926864 | A | G | 0.813 | 1.37E-04 | 44.40 | 0.017 | 0.003 | 2.68E-11 |
| rs10187101 | T | C | 0.712 | 1.36E-04 | 44.39 | -0.017 | 0.003 | 2.69E-11 |
| rs111610668 | G | A | 0.852 | 1.36E-04 | 44.19 | -0.017 | 0.003 | 2.99E-11 |
| rs12103006 | A | G | 0.533 | 1.35E-04 | 44.02 | -0.017 | 0.003 | 3.25E-11 |
| rs2730806 | T | A | 0.439 | 1.35E-04 | 43.96 | 0.016 | 0.002 | 3.35E-11 |
| rs7035637 | A | G | 0.667 | 1.35E-04 | 43.84 | 0.019 | 0.003 | 3.57E-11 |
| rs8074454 | C | G | 0.706 | 1.35E-04 | 43.74 | 0.017 | 0.003 | 3.75E-11 |
| rs72995085 | C | T | 0.909 | 1.34E-04 | 43.73 | -0.021 | 0.003 | 3.77E-11 |
| rs704061 | C | T | 0.501 | 1.34E-04 | 43.64 | 0.016 | 0.002 | 3.94E-11 |
| rs11679338 | C | T | 0.716 | 1.34E-04 | 43.52 | -0.017 | 0.003 | 4.20E-11 |
| rs12739999 | A | G | 0.616 | 1.32E-04 | 43.00 | 0.022 | 0.003 | 5.46E-11 |
| rs7942037 | C | G | 0.711 | 1.32E-04 | 42.94 | -0.017 | 0.003 | 5.64E-11 |
| rs7308188 | C | T | 0.554 | 1.32E-04 | 42.92 | -0.019 | 0.003 | 5.70E-11 |
| rs4500930 | T | C | 0.632 | 1.32E-04 | 42.90 | 0.017 | 0.003 | 5.75E-11 |
| rs7822494 | C | T | 0.549 | 1.31E-04 | 42.65 | -0.016 | 0.002 | 6.53E-11 |
| rs34811474 | A | G | 0.926 | 1.31E-04 | 42.53 | -0.019 | 0.003 | 6.96E-11 |
| rs684214 | T | C | 0.770 | 1.30E-04 | 42.17 | 0.018 | 0.003 | 8.35E-11 |
| rs13192865 | A | G | 0.813 | 1.29E-04 | 42.08 | -0.018 | 0.003 | 8.76E-11 |
| rs67463976 | C | G | 0.511 | 1.29E-04 | 42.00 | 0.016 | 0.003 | 9.11E-11 |
| rs7165759 | A | G | 0.728 | 1.28E-04 | 41.78 | -0.017 | 0.003 | 1.02E-10 |
| rs74934567 | G | A | 0.882 | 1.28E-04 | 41.54 | -0.022 | 0.003 | 1.16E-10 |
| rs12335914 | C | G | 0.616 | 1.27E-04 | 41.44 | 0.016 | 0.002 | 1.22E-10 |
| rs3791687 | T | A | 0.692 | 1.27E-04 | 41.40 | 0.019 | 0.003 | 1.24E-10 |
| rs114067739 | A | C | 0.975 | 1.26E-04 | 40.84 | -0.037 | 0.006 | 1.66E-10 |
| rs9843340 | C | T | 0.931 | 1.25E-04 | 40.74 | -0.022 | 0.003 | 1.74E-10 |
| rs112108364 | G | T | 0.860 | 1.21E-04 | 39.51 | 0.017 | 0.003 | 3.27E-10 |
| rs217669 | C | T | 0.642 | 1.21E-04 | 39.48 | 0.017 | 0.003 | 3.32E-10 |
| rs55769038 | G | A | 0.372 | 1.21E-04 | 39.44 | -0.016 | 0.003 | 3.38E-10 |
| rs13075615 | T | C | 0.931 | 1.20E-04 | 39.13 | -0.022 | 0.003 | 3.97E-10 |
| rs254024 | T | G | 0.583 | 1.20E-04 | 39.02 | 0.016 | 0.002 | 4.19E-10 |
| rs10789334 | A | G | 0.915 | 1.20E-04 | 39.00 | -0.018 | 0.003 | 4.24E-10 |
| rs6433243 | T | C | 0.649 | 1.19E-04 | 38.86 | 0.016 | 0.003 | 4.56E-10 |
| rs13263674 | G | A | 0.861 | 1.19E-04 | 38.82 | 0.017 | 0.003 | 4.65E-10 |
| rs215628 | C | T | 0.398 | 1.17E-04 | 38.17 | 0.016 | 0.003 | 6.48E-10 |
| rs1474518 | C | T | 0.761 | 1.17E-04 | 38.13 | -0.018 | 0.003 | 6.62E-10 |
| rs2962082 | A | G | 0.614 | 1.17E-04 | 38.10 | -0.015 | 0.002 | 6.72E-10 |
| rs7654647 | T | A | 0.700 | 1.17E-04 | 38.06 | 0.016 | 0.003 | 6.86E-10 |
| rs1724557 | C | A | 0.372 | 1.17E-04 | 38.06 | 0.016 | 0.003 | 6.87E-10 |
| rs9925945 | C | A | 0.782 | 1.17E-04 | 38.01 | -0.017 | 0.003 | 7.04E-10 |
| rs2667761 | C | T | 0.492 | 1.17E-04 | 37.95 | -0.016 | 0.003 | 7.27E-10 |
| rs809955 | A | G | 0.646 | 1.17E-04 | 37.90 | -0.016 | 0.003 | 7.45E-10 |
| rs10773302 | G | T | 0.803 | 1.16E-04 | 37.85 | -0.017 | 0.003 | 7.66E-10 |
| rs12001634 | A | T | 0.531 | 1.16E-04 | 37.79 | -0.016 | 0.003 | 7.88E-10 |
| rs11126734 | A | C | 0.654 | 1.16E-04 | 37.77 | -0.015 | 0.002 | 7.97E-10 |
| rs35972789 | A | C | 0.990 | 1.15E-04 | 37.30 | -0.040 | 0.006 | 1.01E-09 |
| rs11776713 | C | T | 0.626 | 1.15E-04 | 37.25 | -0.015 | 0.002 | 1.04E-09 |
| rs4148866 | T | C | 0.603 | 1.13E-04 | 36.84 | 0.015 | 0.003 | 1.28E-09 |
| rs4399192 | G | T | 0.726 | 1.11E-04 | 36.17 | 0.018 | 0.003 | 1.81E-09 |
| rs2499468 | C | A | 0.775 | 1.11E-04 | 36.00 | -0.016 | 0.003 | 1.98E-09 |
| rs13097150 | T | C | 0.699 | 1.11E-04 | 35.94 | 0.015 | 0.003 | 2.03E-09 |
| rs362307 | T | C | 0.972 | 1.10E-04 | 35.93 | 0.029 | 0.005 | 2.04E-09 |
| rs1762509 | A | G | 0.775 | 1.10E-04 | 35.79 | 0.016 | 0.003 | 2.20E-09 |
| rs2020942 | T | C | 0.745 | 1.10E-04 | 35.72 | 0.015 | 0.003 | 2.28E-09 |
| rs3803253 | A | G | 0.797 | 1.10E-04 | 35.62 | -0.016 | 0.003 | 2.39E-09 |
| rs778094 | G | A | 0.342 | 1.09E-04 | 35.48 | 0.015 | 0.003 | 2.57E-09 |
| rs78719460 | A | G | 0.864 | 1.09E-04 | 35.29 | 0.016 | 0.003 | 2.83E-09 |
| rs1229984 | T | C | 0.841 | 1.09E-04 | 35.29 | -0.050 | 0.008 | 2.84E-09 |
| rs62183012 | C | T | 0.879 | 1.08E-04 | 35.20 | -0.016 | 0.003 | 2.97E-09 |
| rs12435171 | G | A | 0.388 | 1.08E-04 | 35.19 | 0.016 | 0.003 | 2.98E-09 |
| rs9832402 | G | A | 0.685 | 1.08E-04 | 35.19 | -0.017 | 0.003 | 2.99E-09 |
| rs61903695 | G | A | 0.854 | 1.08E-04 | 35.16 | 0.017 | 0.003 | 3.04E-09 |
| rs2472297 | T | C | 0.934 | 1.08E-04 | 35.13 | 0.017 | 0.003 | 3.08E-09 |
| rs754635 | C | G | 0.766 | 1.08E-04 | 35.01 | -0.023 | 0.004 | 3.28E-09 |
| rs2102278 | G | A | 0.455 | 1.07E-04 | 34.90 | 0.016 | 0.003 | 3.48E-09 |
| rs117176448 | G | C | 0.963 | 1.07E-04 | 34.79 | 0.025 | 0.004 | 3.67E-09 |
| rs4562625 | C | G | 0.666 | 1.07E-04 | 34.74 | 0.015 | 0.003 | 3.76E-09 |
| rs9569934 | T | C | 0.773 | 1.06E-04 | 34.61 | -0.019 | 0.003 | 4.04E-09 |
| rs73213484 | T | A | 0.839 | 1.06E-04 | 34.56 | -0.021 | 0.004 | 4.13E-09 |
| rs1559678 | C | T | 0.441 | 1.06E-04 | 34.52 | 0.015 | 0.003 | 4.22E-09 |
| rs55911231 | T | C | 0.559 | 1.06E-04 | 34.51 | 0.015 | 0.003 | 4.25E-09 |
| rs60377014 | T | C | 0.882 | 1.06E-04 | 34.38 | -0.020 | 0.003 | 4.53E-09 |
| rs2804477 | A | G | 0.849 | 1.05E-04 | 34.00 | 0.021 | 0.004 | 5.52E-09 |
| rs7586854 | T | C | 0.398 | 1.04E-04 | 33.97 | -0.014 | 0.002 | 5.60E-09 |
| rs12200046 | T | C | 0.932 | 1.04E-04 | 33.87 | 0.022 | 0.004 | 5.90E-09 |
| rs2799465 | C | T | 0.711 | 1.04E-04 | 33.83 | 0.021 | 0.004 | 6.02E-09 |
| rs3774063 | T | C | 0.959 | 1.03E-04 | 33.64 | 0.024 | 0.004 | 6.63E-09 |
| rs62473743 | A | G | 0.734 | 1.03E-04 | 33.59 | 0.020 | 0.003 | 6.82E-09 |
| rs3759094 | T | C | 0.733 | 1.03E-04 | 33.52 | -0.015 | 0.003 | 7.05E-09 |
| rs12409875 | A | G | 0.644 | 1.03E-04 | 33.48 | -0.014 | 0.002 | 7.21E-09 |
| rs247975 | T | C | 0.631 | 1.03E-04 | 33.41 | -0.014 | 0.002 | 7.46E-09 |
| rs3826408 | T | C | 0.613 | 1.02E-04 | 33.29 | 0.014 | 0.002 | 7.95E-09 |
| rs8103728 | C | G | 0.618 | 1.02E-04 | 33.27 | -0.015 | 0.003 | 8.02E-09 |
| rs61537964 | G | C | 0.857 | 1.02E-04 | 33.25 | -0.023 | 0.004 | 8.11E-09 |
| rs62413414 | T | C | 0.927 | 1.02E-04 | 33.09 | 0.020 | 0.003 | 8.78E-09 |
| rs11917587 | A | G | 0.484 | 1.02E-04 | 33.06 | 0.014 | 0.003 | 8.94E-09 |
| rs34431565 | T | G | 0.967 | 1.02E-04 | 33.02 | -0.034 | 0.006 | 9.14E-09 |
| rs17589357 | C | T | 0.937 | 1.01E-04 | 32.72 | -0.020 | 0.003 | 1.07E-08 |
| rs62024481 | T | C | 0.845 | 1.00E-04 | 32.60 | -0.018 | 0.003 | 1.13E-08 |
| rs1446585 | G | A | 0.190 | 9.99E-05 | 32.48 | -0.017 | 0.003 | 1.21E-08 |
| rs7773094 | C | T | 0.738 | 9.99E-05 | 32.48 | -0.018 | 0.003 | 1.21E-08 |
| rs7324067 | T | C | 0.775 | 9.97E-05 | 32.42 | -0.017 | 0.003 | 1.24E-08 |
| rs2537621 | C | G | 0.676 | 9.95E-05 | 32.34 | 0.015 | 0.003 | 1.29E-08 |
| rs10510025 | T | C | 0.634 | 9.92E-05 | 32.25 | 0.016 | 0.003 | 1.36E-08 |
| rs7849553 | C | A | 0.593 | 9.89E-05 | 32.16 | 0.014 | 0.002 | 1.42E-08 |
| rs4807179 | G | A | 0.394 | 9.88E-05 | 32.12 | -0.015 | 0.003 | 1.45E-08 |
| rs6536575 | T | C | 0.494 | 9.86E-05 | 32.08 | -0.014 | 0.002 | 1.48E-08 |
| rs148168215 | T | A | 0.984 | 9.84E-05 | 32.01 | -0.053 | 0.009 | 1.54E-08 |
| rs9304665 | T | A | 0.521 | 9.78E-05 | 31.82 | -0.017 | 0.003 | 1.70E-08 |
| rs7021721 | C | G | 0.723 | 9.77E-05 | 31.77 | -0.015 | 0.003 | 1.74E-08 |
| rs11173521 | T | G | 0.398 | 9.71E-05 | 31.57 | 0.014 | 0.003 | 1.93E-08 |
| rs12632423 | A | G | 0.840 | 9.64E-05 | 31.36 | -0.023 | 0.004 | 2.14E-08 |
| rs4842920 | T | G | 0.882 | 9.58E-05 | 31.16 | -0.015 | 0.003 | 2.38E-08 |
| rs7724430 | A | C | 0.470 | 9.52E-05 | 30.95 | 0.014 | 0.003 | 2.65E-08 |
| rs4872376 | C | T | 0.552 | 9.51E-05 | 30.93 | -0.014 | 0.002 | 2.67E-08 |
| rs58120873 | A | G | 0.938 | 9.51E-05 | 30.93 | -0.025 | 0.004 | 2.67E-08 |
| rs17239176 | C | T | 0.896 | 9.47E-05 | 30.81 | -0.017 | 0.003 | 2.85E-08 |
| rs7864091 | A | G | 0.828 | 9.46E-05 | 30.76 | 0.019 | 0.003 | 2.92E-08 |
| rs73033486 | A | G | 0.905 | 9.45E-05 | 30.73 | 0.021 | 0.004 | 2.97E-08 |
| rs17682873 | T | C | 0.924 | 9.43E-05 | 30.66 | 0.020 | 0.004 | 3.07E-08 |
| rs4809221 | G | A | 0.652 | 9.41E-05 | 30.62 | -0.015 | 0.003 | 3.15E-08 |
| rs916289 | T | C | 0.714 | 9.39E-05 | 30.55 | -0.014 | 0.002 | 3.26E-08 |
| rs496072 | T | C | 0.295 | 9.38E-05 | 30.50 | 0.014 | 0.003 | 3.35E-08 |
| rs2448916 | A | C | 0.472 | 9.34E-05 | 30.36 | -0.014 | 0.003 | 3.60E-08 |
| rs111363146 | C | T | 0.933 | 9.33E-05 | 30.33 | 0.020 | 0.004 | 3.64E-08 |
| rs11896591 | G | A | 0.638 | 9.33E-05 | 30.33 | 0.014 | 0.002 | 3.65E-08 |
| rs264932 | A | G | 0.825 | 9.32E-05 | 30.31 | 0.014 | 0.003 | 3.69E-08 |
| rs4419475 | T | A | 0.418 | 9.32E-05 | 30.30 | 0.014 | 0.003 | 3.71E-08 |
| rs10057588 | G | A | 0.692 | 9.30E-05 | 30.23 | -0.015 | 0.003 | 3.84E-08 |
| rs57241669 | G | A | 0.812 | 9.28E-05 | 30.18 | -0.026 | 0.005 | 3.93E-08 |
| rs9512696 | A | G | 0.471 | 9.28E-05 | 30.16 | -0.014 | 0.003 | 3.97E-08 |
| rs329124 | G | A | 0.538 | 9.24E-05 | 30.04 | -0.014 | 0.003 | 4.22E-08 |
| rs59066241 | G | T | 0.767 | 9.21E-05 | 29.95 | 0.021 | 0.004 | 4.42E-08 |
| rs11161044 | G | C | 0.659 | 9.20E-05 | 29.91 | -0.017 | 0.003 | 4.52E-08 |
| rs12101386 | T | G | 0.858 | 9.14E-05 | 29.72 | -0.016 | 0.003 | 4.98E-08 |
| rs7788950 | A | G | 0.811 | 9.14E-05 | 29.72 | -0.017 | 0.003 | 4.99E-08 |

Abbreviations: SNP, single nucleotide polymorphism; EA, effect alleles; NEA, non-effect alleles; EAF, effect allele frequency; SE, standard error. R^2^, the variance explained by the SNP, calculated as R^2^ = 2 × Beta^2^ × EAF × (1 – EAF )^2^ / (2 × Beta^2^ × EAF × (1 − EAF)^2^ + 2 × SE^2^ × EAF × (1 − EAF) × N), where N stands for the sample size of 325,153; F, the F-statistic, was calculated as follows: F = (N − 2) × R^2^ / (1 − R^2^).

**Table S2.** Proxy SNPs for visceral adipose tissue-associated SNPs which were not available in the outcome datasets.

| **Traits** | **SNP** | **EA** | **NEA** | **Beta** | **SE** | **P-value** | **Proxy SNP** | **r^2^** |
| --- | --- | --- | --- | --- | --- | --- | --- | --- |
| COVID-19 hospitalization | rs113211479 | A | G | 0.024 | 0.003 | 3.70E-22 | rs12419507 | 1.00 |
| COVID-19 susceptibility | rs113211479 | A | G | 0.024 | 0.003 | 3.70E-22 | rs12419507 | 1.00 |
| COVID-19 severity | rs113211479 | A | G | 0.024 | 0.003 | 3.70E-22 | rs12419507 | 1.00 |
| COVID-19 severity | rs669696 | A | C | -0.024 | 0.003 | 6.15E-22 | rs12925700 | 0.83 |
| COVID-19 severity | rs7654647 | T | A | 0.016 | 0.003 | 6.86E-10 | NA | NA |
| COVID-19 severity | rs9358912 | T | G | -0.025 | 0.003 | 1.22E-18 | NA | NA |
| COVID-19 hospitalization (European, leave UKB) | rs113211479 | A | G | 0.024 | 0.003 | 3.70E-22 | rs12419507 | 1.00 |
| COVID-19 hospitalization (European, leave UKB) | rs35972789 | A | C | -0.040 | 0.006 | 1.01E-09 | NA | NA |
| COVID-19 hospitalization (European, leave UKB) | rs55911231 | T | C | 0.015 | 0.003 | 4.25E-09 | rs9554333 | 0.96 |
| COVID-19 hospitalization (European, leave UKB) | rs9358912 | T | G | -0.025 | 0.003 | 1.22E-18 | NA | NA |
| COVID-19 susceptibility (European, leave UKB) | rs10057588 | G | A | -0.015 | 0.003 | 3.84E-08 | rs6864955 | 0.97 |
| COVID-19 susceptibility (European, leave UKB) | rs11173521 | T | G | 0.014 | 0.003 | 1.93E-08 | rs11173526 | 0.98 |
| COVID-19 susceptibility (European, leave UKB) | rs113211479 | A | G | 0.024 | 0.003 | 3.70E-22 | rs12419507 | 1.00 |
| COVID-19 susceptibility (European, leave UKB) | rs2730806 | T | A | 0.016 | 0.002 | 3.35E-11 | rs2733278 | 1.00 |
| COVID-19 susceptibility (European, leave UKB) | rs55911231 | T | C | 0.015 | 0.003 | 4.25E-09 | rs9554333 | 0.96 |
| COVID-19 susceptibility (European, leave UKB) | rs669696 | A | C | -0.024 | 0.003 | 6.15E-22 | rs12925700 | 0.83 |
| COVID-19 susceptibility (European, leave UKB) | rs7654647 | T | A | 0.016 | 0.003 | 6.86E-10 | NA | NA |
| COVID-19 severity (European, leave UKB) | rs10740991 | G | C | 0.026 | 0.003 | 1.33E-20 | NA | NA |
| COVID-19 severity (European, leave UKB) | rs10773302 | G | T | -0.017 | 0.003 | 7.66E-10 | rs61954972 | 0.95 |
| COVID-19 severity (European, leave UKB) | rs10789334 | A | G | -0.018 | 0.003 | 4.24E-10 | rs61765616 | 0.88 |
| COVID-19 severity (European, leave UKB) | rs111610668 | G | A | -0.017 | 0.003 | 2.99E-11 | rs147410092 | 0.97 |
| COVID-19 severity (European, leave UKB) | rs11173521 | T | G | 0.014 | 0.003 | 1.93E-08 | rs2723741 | 0.93 |
| COVID-19 severity (European, leave UKB) | rs113211479 | A | G | 0.024 | 0.003 | 3.70E-22 | rs12419507 | 1.00 |
| COVID-19 severity (European, leave UKB) | rs11776713 | C | T | -0.015 | 0.002 | 1.04E-09 | rs17648656 | 1.00 |
| COVID-19 severity (European, leave UKB) | rs12103006 | A | G | -0.017 | 0.003 | 3.25E-11 | NA | NA |
| COVID-19 severity (European, leave UKB) | rs12200046 | T | C | 0.022 | 0.004 | 5.90E-09 | rs10499276 | 1.00 |
| COVID-19 severity (European, leave UKB) | rs1229984 | T | C | -0.050 | 0.008 | 2.84E-09 | NA | NA |
| COVID-19 severity (European, leave UKB) | rs12409875 | A | G | -0.014 | 0.002 | 7.21E-09 | rs3001347 | 0.90 |
| COVID-19 severity (European, leave UKB) | rs13263674 | G | A | 0.017 | 0.003 | 4.65E-10 | rs10088445 | 0.99 |
| COVID-19 severity (European, leave UKB) | rs13393304 | A | G | -0.044 | 0.003 | 6.12E-42 | rs6548239 | 1.00 |
| COVID-19 severity (European, leave UKB) | rs1454687 | C | G | 0.023 | 0.002 | 1.49E-20 | rs930813 | 0.85 |
| COVID-19 severity (European, leave UKB) | rs1762509 | A | G | 0.016 | 0.003 | 2.20E-09 | rs6669831 | 0.98 |
| COVID-19 severity (European, leave UKB) | rs1834144 | A | C | -0.018 | 0.003 | 1.72E-12 | rs1424394 | 1.00 |
| COVID-19 severity (European, leave UKB) | rs2253310 | C | G | -0.021 | 0.003 | 3.16E-16 | NA | NA |
| COVID-19 severity (European, leave UKB) | rs2307111 | C | T | -0.026 | 0.003 | 1.08E-24 | rs16872768 | 0.90 |
| COVID-19 severity (European, leave UKB) | rs2499468 | C | A | -0.016 | 0.003 | 1.98E-09 | rs1266910 | 0.99 |
| COVID-19 severity (European, leave UKB) | rs2537621 | C | G | 0.015 | 0.003 | 1.29E-08 | rs9691558 | 0.87 |
| COVID-19 severity (European, leave UKB) | rs2667761 | C | T | -0.016 | 0.003 | 7.27E-10 | rs867917 | 1.00 |
| COVID-19 severity (European, leave UKB) | rs2730806 | T | A | 0.016 | 0.002 | 3.35E-11 | rs2733278 | 1.00 |
| COVID-19 severity (European, leave UKB) | rs2926614 | T | C | -0.022 | 0.003 | 4.16E-12 | rs830783 | 0.92 |
| COVID-19 severity (European, leave UKB) | rs34811474 | A | G | -0.019 | 0.003 | 6.96E-11 | NA | NA |
| COVID-19 severity (European, leave UKB) | rs3787075 | G | C | 0.018 | 0.003 | 1.26E-11 | NA | NA |
| COVID-19 severity (European, leave UKB) | rs3791687 | T | A | 0.019 | 0.003 | 1.24E-10 | NA | NA |
| COVID-19 severity (European, leave UKB) | rs40067 | A | G | -0.024 | 0.003 | 1.59E-13 | rs158186 | 1.00 |
| COVID-19 severity (European, leave UKB) | rs4073582 | A | G | -0.018 | 0.003 | 1.20E-11 | rs524859 | 0.99 |
| COVID-19 severity (European, leave UKB) | rs4148866 | T | C | 0.015 | 0.003 | 1.28E-09 | rs3897102 | 0.87 |
| COVID-19 severity (European, leave UKB) | rs4482463 | C | A | 0.036 | 0.005 | 1.68E-14 | NA | NA |
| COVID-19 severity (European, leave UKB) | rs4558773 | A | G | 0.017 | 0.003 | 2.24E-11 | rs35776246 | 1.00 |
| COVID-19 severity (European, leave UKB) | rs4562625 | C | G | 0.015 | 0.003 | 3.76E-09 | NA | NA |
| COVID-19 severity (European, leave UKB) | rs4809221 | G | A | -0.015 | 0.003 | 3.15E-08 | rs6011066 | 1.00 |
| COVID-19 severity (European, leave UKB) | rs539515 | C | A | 0.038 | 0.003 | 1.43E-35 | rs516636 | 0.94 |
| COVID-19 severity (European, leave UKB) | rs56094641 | G | A | 0.065 | 0.003 | 3.82E-145 | rs62048402 | 0.98 |
| COVID-19 severity (European, leave UKB) | rs61813293 | T | G | 0.024 | 0.004 | 1.93E-11 | NA | NA |
| COVID-19 severity (European, leave UKB) | rs62024481 | T | C | -0.018 | 0.003 | 1.13E-08 | NA | NA |
| COVID-19 severity (European, leave UKB) | rs62104473 | T | C | 0.019 | 0.003 | 3.50E-13 | rs17513613 | 1.00 |
| COVID-19 severity (European, leave UKB) | rs6739755 | A | G | 0.023 | 0.003 | 3.95E-19 | rs6730325 | 0.99 |
| COVID-19 severity (European, leave UKB) | rs7021721 | C | G | -0.015 | 0.003 | 1.74E-08 | NA | NA |
| COVID-19 severity (European, leave UKB) | rs719802 | T | C | 0.017 | 0.003 | 7.84E-12 | NA | NA |
| COVID-19 severity (European, leave UKB) | rs7324067 | T | C | -0.017 | 0.003 | 1.24E-08 | rs9540259 | 0.89 |
| COVID-19 severity (European, leave UKB) | rs7498665 | G | A | 0.027 | 0.003 | 1.83E-26 | rs11863370 | 1.00 |
| COVID-19 severity (European, leave UKB) | rs754635 | C | G | -0.023 | 0.004 | 3.28E-09 | NA | NA |
| COVID-19 severity (European, leave UKB) | rs76040172 | A | G | -0.045 | 0.006 | 2.60E-16 | NA | NA |
| COVID-19 severity (European, leave UKB) | rs7724430 | A | C | 0.014 | 0.003 | 2.65E-08 | NA | NA |
| COVID-19 severity (European, leave UKB) | rs7773094 | C | T | -0.018 | 0.003 | 1.21E-08 | rs9492242 | 1.00 |
| COVID-19 severity (European, leave UKB) | rs7864091 | A | G | 0.019 | 0.003 | 2.92E-08 | rs7040568 | 0.99 |
| COVID-19 severity (European, leave UKB) | rs7893571 | G | T | -0.018 | 0.003 | 1.17E-11 | NA | NA |
| COVID-19 severity (European, leave UKB) | rs7942037 | C | G | -0.017 | 0.003 | 5.64E-11 | NA | NA |
| COVID-19 severity (European, leave UKB) | rs8103728 | C | G | -0.015 | 0.003 | 8.02E-09 | NA | NA |
| COVID-19 severity (European, leave UKB) | rs9320823 | T | C | -0.023 | 0.003 | 4.92E-20 | rs6938973 | 1.00 |
| COVID-19 severity (European, leave UKB) | rs9358912 | T | G | -0.025 | 0.003 | 1.22E-18 | NA | NA |
| COVID-19 severity (European, leave UKB) | rs9512696 | A | G | -0.014 | 0.003 | 3.97E-08 | rs1218824 | 1.00 |
| COVID-19 severity (European, leave UKB) | rs9522285 | A | G | 0.019 | 0.003 | 9.61E-14 | rs9522289 | 1.00 |
| COVID-19 severity (European, leave UKB) | rs9569934 | T | C | -0.019 | 0.003 | 4.04E-09 | rs78388442 | 1.00 |

Abbreviations: SNP, single nucleotide polymorphism; EA, effect alleles; NEA, non-effect alleles; SE, standard error; COVID-19, Coronavirus Disease 2019; UKB, UK Biobank.

**Table S3.** Power calculation for two-sample MR analyses of visceral adipose tissue on COVID-19 outcomes.

| **Outcome traits** | **Sample size** | **K** | **R^2^** | **Required OR** |
| --- | --- | --- | --- | --- |
| COVID-19 susceptibility | 2,586,691 | 0.04353516 | 0.037 | 1.05 |
| COVID-19 hospitalization | 2,085,803 | 0.01163772 | 0.037 | 1.10 |
| COVID-19 severity | 1,010,654 | 0.00868645 | 0.037 | 1.16 |

Abbreviations: K, proportion of cases in the outcome study; R^2^, the variance explained by the visceral adipose tissue-related instrumental variables; OR, odds ratio.

**Table S4.** Heterogeneity and pleiotropy assessment in supplementary analyses.

| Outcomes | SNPs | P_Q-statistics_ | I^2^ (%) | P_intercept_ | P_MR-PRESSO global test_ | Outlier |
| --- | --- | --- | --- | --- | --- | --- |
| COVID-19 susceptibility (European, leave UKB) | 220 | 4.69E-04 | 25.7 | 0.803 | <0.001 | rs13135092 |
| COVID-19 hospitalization (European, leave UKB) | 219 | 0.007 | 20.1 | 0.755 | 0.007 | rs1446585 |
| COVID-19 severity (European, leave UKB) | 201 | 0.047 | 14.8 | 0.735 | 0.052 | - |

Abbreviations: SNPs, single nucleotide polymorphisms; MR-PRESSO, Mendelian randomization pleiotropy residual sum and outlier; COVID-19, Coronavirus Disease 2019; UKB, UK Biobank.

**Table S5.** Leave-one-out analysis of association between genetically predicted visceral adipose tissue and the risk of COVID-19.

| Outcomes | IVW estimate_all_^a^ | P-value | IVW estimate_min_^b^ | P-value | IVW estimate_max_^c^ | P-value |
| --- | --- | --- | --- | --- | --- | --- |
| COVID-19 susceptibility | 1.13 | 4.37E-12 | 1.13 | 4.85E-12 | 1.14 | 1.09E-12 |
| COVID-19 hospitalization | 1.51 | 4.14E-20 | 1.50 | 9.84E-20 | 1.54 | 1.40E-20 |
| COVID-19 severity | 1.58 | 7.34E-11 | 1.56 | 1.68E-10 | 1.60 | 7.83E-11 |
| COVID-19 susceptibility (European, leave UKB) | 1.09 | 4.07E-05 | 1.08 | 7.36E-05 | 1.10 | 1.22E-05 |
| COVID-19 hospitalization (European, leave UKB) | 1.36 | 1.03E-07 | 1.35 | 2.14E-07 | 1.40 | 2.13E-08 |
| COVID-19 severity (European, leave UKB) | 1.55 | 3.97E-04 | 1.51 | 6.91E-04 | 1.60 | 1.54E-04 |

Abbreviations: IVW, inverse-variance weighted; COVID-19, Coronavirus Disease 2019; UKB, UK Biobank.

^a^: the value of IVW estimate with all SNP included;

^b^: the minimum value of IVW estimate;

^c^: the maximum value of IVW estimate.
